# Supplementary material for: Ser/Thr kinase Trc controls neurite outgrowth in Drosophila by modulating microtubule-microtubule sliding
Source: eLife. 2020 Feb 5;9:e52009. doi: 10.7554/eLife.52009 (PMC7021487; doi:10.7554/eLife.52009)
Supplement: Supplementary file 1. [file elife-52009-supp1.docx]

| Supplementary table 1. Primer sequences for dsRNA generation. | |
| --- | --- |
| Trc Fwd | GCTTGAAGGTTGCCGCACTTTGC |
| Trc Rev | GGGTATTTCGCTGCTGCCCAATAAG |
| Trc 3’ UTR fwd | GGTTGCCGCACTTTGCCACCC |
| Trc 3’ UTR rev | GCGTTTAACCTAGCCCGAGGCG |
| Pavarotti fwd | AAATCCGTAACGAAACTAACCG |
| Pavarotti rev | ACAACTGCTCTTGGCAGATACC |
| Pavarotti 3’UTR fwd | AAATGACTCAGCGTGGAATTCTC |
| Pavarotti 3’UTR rev | CAGTATATGCGCGTAATTCACTTTAT |
| Pavarotti 5’UTR fwd | TCGGTCACTCTAAAACCAAGCGTG |
| Pavarotti 5’UTR rev | TCGGTCACTCTAAAACCAAGCGTG |
| Fry fwd | GCCCAGAACGGTGCCAGTCC |
| Fry rev | CCGTGCACGACATCCTGACGCC |
| 14-3-3 epsilon fwd | GGTGGAGGCCATGAAGAAG GTCGC |
| 14-3-3 epsilon rev | CGGATGGGGTGTGTTGGTGG |
| 14-3-3 zeta fwd | CTGGACACACTGAACGAGG ACTCCTA |
| 14-3-3 zeta rev | CATTTGCTTAGTTGTTTGGTTA GTTGTCGCC |
